# Within-participant statistics for cognitive science

**DOI:** 10.1016/j.tics.2022.05.008

**Published:** 2022-08

**Authors:** Robin A.A. Ince, Jim W. Kay, Philippe G. Schyns

**Affiliations:** 1 School of Psychology and Neuroscience, University of Glasgow, Glasgow, UK; 2 Department of Statistics, University of Glasgow, Glasgow, UK

**Keywords:** statistics, prevalence, replicability, inference, within-participant, individual differences

## Abstract

Experimental studies in cognitive science typically focus on the population average effect. An alternative is to test each individual participant and then quantify the proportion of the population that would show the effect: the prevalence, or participant replication probability. We argue that this approach has conceptual and practical advantages.

The goal of a scientific experiment is to learn something about the world. In the cognitive sciences, experiments are typically performed on a sample of participants randomly selected from a **population** (see Glossary, [1]). Statistical methods are used to make a quantitative statement about the population from the results of the experiment.

Many experimental questions pertain to the existence of an effect. For example, whether stimuli of a particular class activate a particular brain region. Typically, researchers address such questions from the perspective of the **population mean**, by applying **null-hypothesis significance testing** (NHST) to determine whether the mean effect is different from zero (statistically significant).

An alternative is to evaluate whether each individual participant demonstrates the effect and then quantify the **population prevalence** – the proportion of the population that would show the effect if they were tested in this experiment [2]. This approach allows reliable scientific knowledge to be obtained through longer experiments with fewer participants, as in psychophysics [3]. However, without the formal generalization to the population provided by prevalence, such results are often dismissed as **case studies**.

## Within-participant statistics and population prevalence

Recent developments allow generalization of within-participant results to the population prevalence, using either frequentist [4] or Bayesian [2] methods (Box 1). Bayesian prevalence is straightforward to apply to any experiment. It requires only that we test the effect of interest separately in each participant, controlling the false positive rate of the within-participant test (e.g., by verifying modeling assumptions or using distribution-free methods). The within-participant test itself can be performed using any statistical or modeling approach (linear or nonlinear, parametric or nonparametric, inferential or predictive). Although our focus here is the human participant, Bayesian prevalence can be directly applied to other organisms (e.g., rodents), models (e.g., deep neural networks), or sampled units (e.g., neurons).

Box 1 **Bayesian prevalence**

Several approaches quantitively summarize within-participant results. Grice *et al.* [11] propose reporting the sample proportion as a person-centered effect size, but this does not provide a formal generalization to the population. Frequentist NHST methods applied to a binomial model can test various hypotheses about the population prevalence (e.g., the global null, that the prevalence is 0, or the majority null, that the prevalence is <0.5, Figure IA) [4,12]. We recently proposed a Bayesian method to estimate the population within-participant replication probability, accounting for the false positive rate of the statistical test [2]. Bayesian prevalence returns a posterior distribution over the population prevalence, given the observed experimental data (Figure IB). From this, we can compute the maximum a posterior (MAP) estimate – the best guess, or most likely value of the population parameter (Figure IC). To quantify the uncertainty of this estimate, we compute Bayesian highest posterior density intervals (HPDIs) for various levels (such as 50% and 96%; Figure IB). These intervals provide the range within which the true population value lies with the specified probability. Bayesian prevalence can also quantify the posterior distribution for the difference in prevalence between different tests performed on the same participants, or between the same test applied to samples of participants from different populations.The posterior prevalence can be calculated for different effect size thresholds (not just *p* = 0.05) [2]. Open source code implementing Bayesian prevalence in Python, Matlab and R is available at https://github.com/robince/bayesian-prevalence. An online web application is available at https://estimate.prevalence.online/.

**Figure I:**
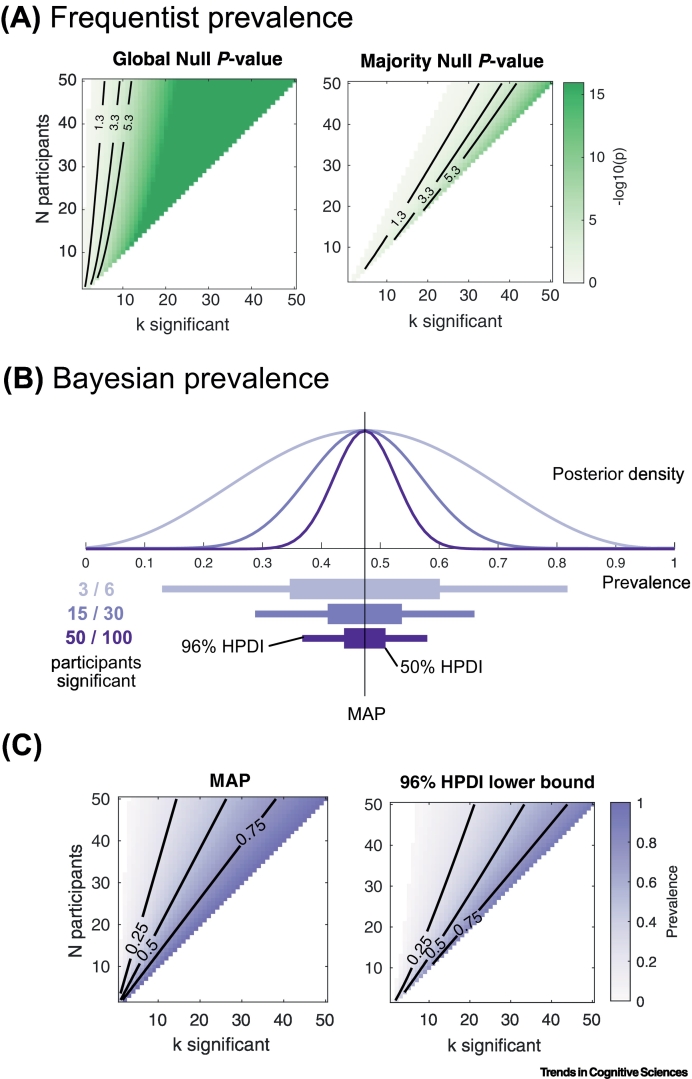
Estimating population prevalence. (A) Frequentist *P* values for the global null hypothesis (left, population prevalence is 0; i.e., no participant shows an effect) and majority null hypothesis (right, population prevalence is <0.5) as a function of the number of significant participants *k* (x axis, within-participant test at α=0.05) in an experiment with *N* participants in total (y axis). The contour with value -log_10_(*p*)=1.3 corresponds to the typical *P*=0.05 significance level. Each subsequent contour shows a 100-fold decrease in the *P* value, that is, 100 times as many exploratory analyses would be required, on average, to see a false-positive result. (B) Bayesian prevalence posterior, MAP estimate, and highest posterior density (HPD) intervals are illustrated. Increasing participant numbers does not change the MAP (black vertical line), but reduces the uncertainty in the estimate [thin line, 96% HPDI; thick line 50% HPDI). (C) MAP estimate (left) and lower edge of 96% HPD interval (right) are shown (axes as A). A lower bound on population prevalence of 0.25 can be obtained from experiments with different numbers of participants (e.g., 4/5, 6/10, 10/20, and 22/50). Considering the *P* value of the global null, these results all offer orders of magnitude stronger evidence of a population-level effect than the standard *P*=0.05 (-log(*p*)=1.3 contour), and therefore are less likely to occur as a false-positive result from researcher degrees of freedom in an exploratory analysis (global null log-*p* respectively 4.5, 5.5, 7.9, and 15.2). Under the global null, a result of 6/10 significant is 10 000 times less likely to occur by chance than the normally accepted standard of evidence for a population mean effect.

Alt-text: Box 1

## Within-participant statistics build in replication

The idea that there may be a problem with common statistical practice in experimental studies of cognition is receiving increased attention. Widely termed the replication crisis, concerns have arisen because many results are not obtained again when the experiment is repeated. NHST of the population mean is usually the only analysis considered when discussing the issues underlying the replication crisis. We highlight two reasons why Bayesian prevalence may be less susceptible to these issues. First, when analyzed separately, each participant provides an independent replication of the experiment. Therefore, Bayesian prevalence has replication built in, and it directly quantifies the population-level, within-participant replication probability. Second, the output of Bayesian prevalence is a posterior distribution for the prevalence of the effect. This provides a graded estimate explicitly including uncertainty. Bayesian prevalence provides a clear quantitative statement about the population within-participant replication probability, which is explicitly linked to the experimental procedure considered. In contrast, NHST reduces an experiment to a binary result (significant or not) whose interpretation involves more challenging logic, often leading to misinterpretation [5] or overinterpretation [6].

## Limitations of Bayesian prevalence

**Figure 1:**
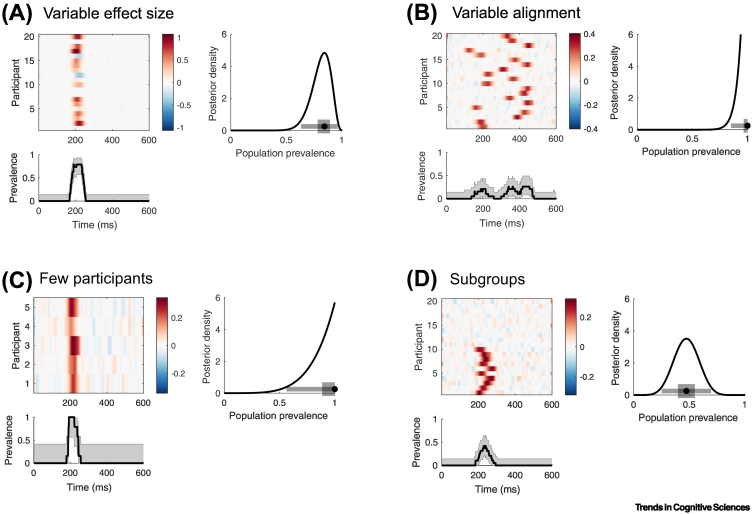
Simulated examples. Simulated examples illustrate how population mean inference and prevalence estimation can differ. Panels A–D show four different simulated electroencephalography experiments. Each panel shows the within-participant mean (upper left), overall population prevalence (right), and prevalence at each time point (lower left). Overall prevalence (right) shows posterior density (black curve) with MAP (circle), 50% and 96% HPDI (thick/thin grey lines), obtained from within-participant inference at *P* = 0.05, Bonferroni corrected over time. Prevalence at each time (lower) shows MAP (black line) and 96% HPDI (grey region). (A) Variable effect size (20 participants). Effect amplitudes were drawn from a normal distribution with high variance. (B) Variable alignment (20 participants). Effects were simulated with fixed amplitude but variable timing. (C) Few participants (5 participants). Effects were simulated with medium amplitude variance and consistent timing. (D) Subgroups (20 participants). Effects were simulated in only ten participants, with fixed amplitude and some timing variability. (A–D) All simulations show no significant population mean effect at any time point (*P* = 0.05, Bonferroni corrected over time). Right: The Bayesian posterior prevalence results provide strong evidence for a population effect in all cases. (A–C) Within-participant replication probability is likely greater than 63%, 85%, and 56% respectively (lower bound 96% HPDI). (D) The population prevalence lies between 25% and 70% (with 96% probability). (A, C, D) The Bayesian prevalence time course (lower left) localizes the population effects in time. (B) Due to the variability in the time of the effect there is no time point with strong evidence of a population effect. Modified from [2]. Code: https://bit.ly/36WIg51.

There are several limitations to Bayesian prevalence. First, it cannot be applied to data from a single participant. In Figure I in Box 1, we show how population prevalence estimates scale with the number of participants. Second, within-participant statistics cannot pool information across individuals as hierarchical models do. Thus, sensitivity to some effects may be decreased. However, prevalence can detect effects that the population mean does not (Figure 1). Third, Bayesian prevalence is currently restricted to effects that are quantifiable within individuals (rather than between-participant research questions), although it can be compared between two populations [2]. Finally, for some effects (e.g., those requiring novelty, learning, or other one-shot interventions) it may be difficult to collect enough data to have sufficient within-participant sensitivity.

## Bayesian prevalence supports new research directions

From cultural psychology to brain stimulation, many fields now recognize the challenge of addressing diversity in cognition, where a single population average cannot provide a full description [7]. For example, the proportion of participants who will respond to a particular brain stimulation protocol is critical to evaluating its practical potential but is not considered in population mean NHST analyses. This argument generalizes to other interventions or biomarkers: the higher bar of evidence set by requiring reliable effects within individuals is a prerequisite for many practical applications.

In neuroimaging, there is renewed interest in the psychophysical approach of longer experiments with fewer subjects [8,9], often combining data over many experimental sessions. Hardware advances such as OPM-MEG and fNIRS allow more participant mobility and more comfortable acquisition of longer sessions. Relatedly, clinical studies of rare conditions often have small numbers of participants who show greater heterogeneity, both of which are problematic for population mean inference (Figure 1). Bayesian prevalence provides a population generalization that is currently missing for both types of small-N studies.

The population mean approach requires alignment of effects across participants, which becomes more challenging as the spatial resolution of imaging techniques increases (e.g., laminar fMRI at 7T), or for invasive methods where electrode positions differ. If the within-participant inference is properly corrected for multiple comparisons, then Bayesian prevalence can be estimated for a broad region of interest without requiring precise overlap of the effect across participants (Figure 1B).

These new recording modalities and approaches require reliable discovery-led exploratory research alongside confirmatory hypothesis testing. Typical NHST has well-documented shortcomings for such exploratory research, where *a priori* effect size estimates (required for power analyses) are difficult to obtain, and power analysis for common multivariate techniques (e.g., cluster methods) is not yet fully developed. Replicating the effect across multiple participants provides a more robust approach and reduces the potential for false positives from researcher degrees of freedom (see Figure I in Box 1).

The development of online experimental platforms has made studies with large numbers of participants more common. One drawback is that with large samples, population mean effects can be detected as statistically significant even when they may be too small to be practically meaningful. Prevalence does not suffer from this drawback. Large numbers of participants allow accurate prevalence estimates, but effects are detected within individual participants and grounded to the experiment considered (e.g., a 10-min experiment vs a 1-h experiment).

It is noteworthy that practical applications of neuroimaging or behavioral biomarkers have been difficult to obtain. One reason for this could be that individuals can differ categorically across many aspects of cognition from behavioral strategy to neural anatomy [3,7]. Another is that the focus on the population mean may have led scientists to study effects with low between-participant variance [10]. However, more variable effects (Figure 1) might be more informative in terms of health and disease outcomes, even though they are less reliable from the population mean perspective.

## Concluding remarks

We argue that an easy-to-adopt epistemological shift in statistical perspective can improve the robustness and interpretability of results in cognitive science and beyond. A focus on the population mean is ubiquitous in cognitive science and, for many, it is synonymous with population generalization. However, for many research questions, effects at the level of the individual participant may be more relevant. Bayesian prevalence explicitly quantifies the within-participant replicability of an experiment, providing a result that is less susceptible to the issues underlying the replication crisis. Prevalence can provide stronger population-level evidence from smaller numbers of participants and is more robust to heterogenous effects (Figure 1). However, estimation of population prevalence and population mean are not mutually exclusive, and they can offer complementary perspectives. Researchers can report within-participant effect sizes and population prevalence, together with an estimate of the population mean, ideally including population variance. Experimental and statistical methods to better describe individual brains, rather than the average brain, might lead to new insights and practical applications.

## Glossary

**Case study**: a descriptive analysis of an individual or group with no statistical generalization to a population. Without generalization, the results pertain only to the participants in the study.
**Null hypothesis significance testing**: starts from a null hypothesis, typically that the population mean is zero. The *P* value quantifies how surprising the observed experimental results would be if that null hypothesis were true. If this is less than a prespecified threshold (usually 0.05), we reject the null hypothesis of zero mean and declare the population mean result to be statistically significant.
**Population**: the larger group from which the participants in an experiment (the sample) were randomly selected. The goal of statistical analysis is to generalize from the sample to the population, which requires a statistical model of the population. Issues around defining the population considered in a study are beyond the scope of this piece.
**Population mean**: the typical approach in cognitive science is to model the population with a Gaussian distribution. The population mean is the true value of the mean parameter of the population Gaussian model.
**Population prevalence**: the population is modeled with a binomial distribution, accounting for the error rates of the within-participant statistical test, with individuals either showing an effect or not. The population prevalence is the binomial proportion parameter of this model. This is the probability of a true positive within-participant replication if the experiment was run on a new randomly sampled participant.
